# Pulmonary tuberculosis infection and CMV reactivation following daratumumab treatment in a patient with relapsed plasmablastic lymphoma

**DOI:** 10.1097/BS9.0000000000000134

**Published:** 2022-08-26

**Authors:** Wenyue Cao, Yuling Wan, Xingcheng Yang, Wei Huang, Jia Wei

**Affiliations:** a Department of Hematology, Tongji Hospital, Tongji Medical College, Huazhong University of Science and Technology, Wuhan, China; b Department of Hematology, Shanxi Bethune Hospital, Shanxi Academy of Medical Sciences, Shanxi Medical University, Shanxi Tongji Hospital, Tongji Medical College, Huazhong University of Science and Technology, Taiyuan, China

**Keywords:** CMV, Daratumumab, Plasmablastic lymphoma, Tuberculosis

## Abstract

Plasmablastic lymphoma (PBL) is an aggressive lymphoma with limited treatment strategies. Tuberculosis (TB) infection poses a high risk for patients with hematologic malignancies, especially those treated with immune agents but were never reported post-daratumumab treatment. Herein, we reported a TB infection in a 57-year-old male diagnosed with HIV-negative PBL receiving daratumumab-based treatment, who showed atypical lung infection and yielded *Mycobacterium tuberculosis* and cytomegalovirus (CMV) in the bronchoalveolar lavage fluid. Anti-TB therapy was administered, and the following daratumumab treatment was complete with good tolerance. In this case, we demonstrated that TB infection might occur after daratumumab therapy, and adequate attention should be paid to atypical symptoms.

## 1. INTRODUCTION

Plasmablastic lymphoma (PBL) is an aggressive lymphoma characterized by early relapse and subsequent chemotherapy resistance.^1^ The immunophenotype of PBL cells is similar to that of plasma cell tumors, positive for CD79a, MUM-1, BLIMP-1, CD38, and CD138.^2^ The response to intense chemotherapy is unsatisfactory, with a median overall survival (OS) of 6 to 19 months.^3,4^ Due to the dismal prognosis, there is no standard of care or treatment guidelines for PBL patients. Thus, we seek additional treatment methods with respect to immunotherapy.

Daratumumab is a first-in-class human IgG1κ monoclonal antibody against CD38 with a direct antitumor and immunomodulatory activities.^5^ Due to the close resemblance of PBL to multiple myeloma, daratumumab seems a potential therapeutic alternative.^6,7^ Several studies have reported the role of daratumumab in the treatment of PBL; however, the outcome is not certain.^8,9^ Currently, only a few treatment options are available for relapsed PBL.

The major adverse events associated with daratumumab combination therapy were thrombocytopenia, neutropenia, and anemia^10^; no significant increase was noted in infectious complications in clinical trials.^11^ However, the reduced immunocompetence associated with this novel monoantibody has gained increasing attention. Herein, we reported a case of relapsed PBL who achieved partial remission (PR) but later developed pulmonary tuberculosis (TB) infection and cytomegalovirus (CMV) reactivation after daratumumab-based treatment.

## 2. CASE PRESENTATION

A 57-year-old male with clinical-stage IVB HIV-negative PBL was referred to the hematology clinic of the Tongji Hospital (Wuhan, China) due to concern of disease progression. He received 6 cycles of CHOP (cyclophosphamide, doxorubicin, vincristine, and prednisone), 2 cycles of R-GemDOx (rituximab, gemcitabine, oxaliplatin, and dexamethasone), and 3 cycles of treatment with temozolomide, ibrutinib, and lenalidomide between May 2019 and October 2020. He had a 9-year history of hypertension but denied any knowledge of the prior infectious disease.

**Figure 1.**
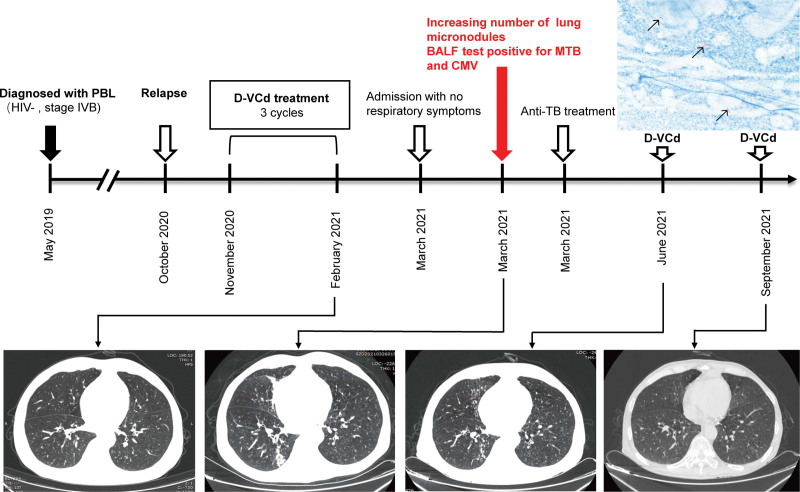
Development of tuberculosis in a patient treated with daratumumab-based therapy for relapsed plasmablastic lymphoma. The male patient with relapsed plasmablastic lymphoma was given D-VCd treatment between November 2020 and February 2021. On March 2021, he was admitted with no respiratory symptoms, while the chest CT showed increasing number of lung micronodules. Fiberoptic bronchoscopy was conducted and bronchoalveolar lavage fluid tested positive for *Mycobacterium tuberculosis* and cytomegalovirus. The positive results of acid-fast staining are indicated by black arrows. On June 2021, the chest CT image showed improved infection after regular anti-tuberculosis and anti-cytomegalovirus therapy, and following D-VCd was given without infectious complications. BALF = bronchoalveolar lavage fluid, CT = computed tomography, D-VCd = daratumumab, bortezomib, cyclophosphamide and dexamethasone, MTB = *Mycobacterium tuberculosis*, PBL = plasmablastic lymphoma, TB = tuberculosis.

**Figure 2.**
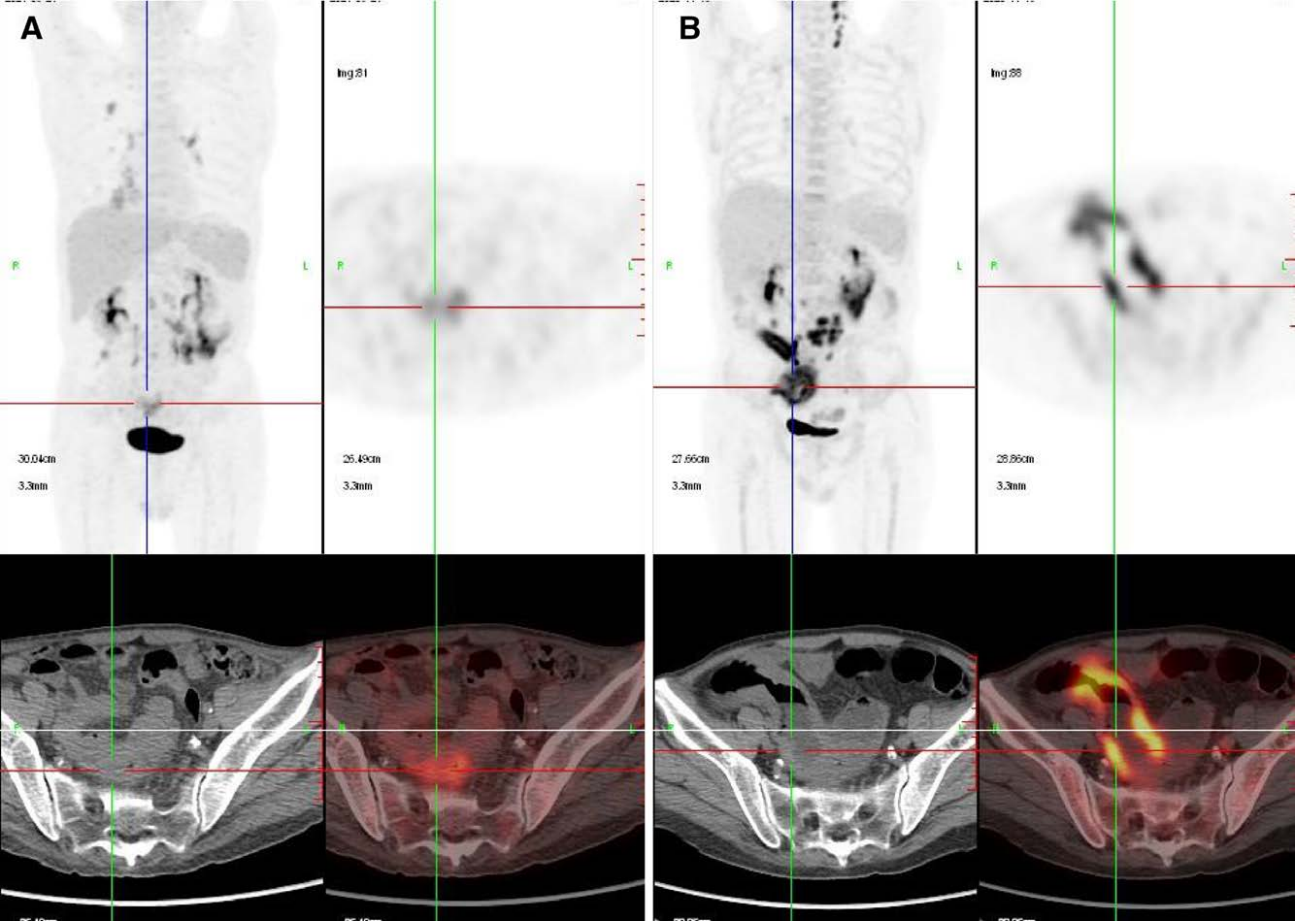
PET/CT images show decreased SUV at retroperitoneal and mesenteric lymph nodes in May 2021 (A) compared to the images in November 2020 (B) after daratumumab-based treatment. The SUV max of retroperitoneal and mesenteric lymph nodes was 16.6 (3.0 cm × 1.7 cm) and decrease to 6.8 (1.8 cm × 1.1 cm). Also, the tumor infiltration in the oropharynx subsided. Partial remission was achieved after 3 cycles of daratumumab-based therapy. PET/CT = positron emission tomography/computed tomography, SUV = standardized uptake value.

To evaluate the disease state, bone marrow puncture and imagological examination were performed. Routine chest computed tomography (CT) showed multiple micronodules (Fig. 1). Multiparameter flow cytometry detected 0.5% PBL cells (CD79a^+^, CD38^+^, CD138^+^, CD20^dim^) with 85.6% Ki-67 expression in the bone marrow. Positron emission tomography/CT (PET/CT) revealed that the involved sites include the right lateral brain ventricle, jejunoileal, retroperitoneal and mesenteric lymph nodes, and the oropharynx (Fig. 2). Immunoprotein electrophoresis detected 2 g/L monoclonal immunoglobulins in the blood serum and urine. All the above suggested disease progression. Due to the low ECOG-PS score, the patient refused stem cell transplantation. Hence, he was treated with daratumumab-based chemotherapy (D-VCd) (daratumumab 16 mg/kg/wk, bortezomib 1.3 g/m^2^ for 4 days/cycle, cyclophosphamide 300 mg/m^2^/wk, and dexamethasone 120 mg/cycle) commencing in December 2020. PR was achieved after 3 cycles of daratumumab-based therapy assessed by CT and minimal residual disease (MRD) was negative in the bone marrow.

In March 2021, he was presented to our hospital for the next course of PBL treatment with no respiratory symptoms. He did not have a cough, fever, expectoration, chest pain, or weight loss. Additionally, he denied a history of TB infection or exposure history. Physical examination revealed scattered crackles in both lungs. The chest CT revealed lung infection with a large number of micronodules (Fig. 1). Laboratory tests revealed microcytic hypochromic anemia (2 × 10^6^ erythrocytes/mm^3^), 11,000 white cells/μL (84% polymorphonuclear cells), 335,000 platelets/μL, and erythrocyte sedimentation rate (ESR) 88 mm/H. Microbial tests, including T-SPOT, sputum smear, and blood cultures, were negative. The lymphocyte subsets indicated B-cell aplasia with hypoglobulinemia, and the patient was in an immunocompromised state (Table 1).

**Table 1:** Immune status before, during, and after daratumumab-based treatment.

Category	November 2020	March 2021	September 2021
Treatment status^*^	Before Dara	During Dara	After Dara
T cell (count/μL)^†^			
CD3+CD19− (range: 955–2860)	1125	421↓	1032
CD3+CD4+ (range: 550–1440)	386↓	118↓	298↓
CD3+CD8+ (range: 320–1250)	671	297↓	692
(Percentage)			
Th/Ts (range: 0.71–2.78)	0.57↓	0.4↓	0.43↓
CD3+CD4+CD28+/Th (range: 84.11–100.00)	62.02↓	52.38↓	57.67↓
CD3+CD8+CD28+/Ts (range: 48.04–77.14)	20.57↓	11.85↓	20.33↓
B cell (count/μL)			
CD3-CD19+ (range: 90–560)	65↓	0↓↓↓	8↓↓
NK cell (count/μL)			
CD3-CD16+CD56+ (range: 150–1100)	93↓	8↓	41↓
Immunoglobulin (g/L)^‡^			
IgM (range: 0.46–3.04)	0.49	0.08↓	0.64
IgG (range: 7.51–15.6)	8.1	4.5↓	9.3
IgA (range: 0.82–4.53)	2.78	<0.07↓	3.85

Dara = daratumumab; D-VCd = daratumumab, bortezomib, cyclophosphamide and dexamethasone, Th = helper T cells, CD3+CD4+, Ts = suppressor T cells, CD3+CD8+.

* The time point representing different treatment status is exhibited. In November 2020, daratumumab is not part of the treatment (Before Dara). In March 2021, the patient has received 3 cycles of D-VCd treatment (During Dara). In September 2021, the patient postponed daratumumab therapy and received continuous anti-tuberculosis and anti-cytomegalovirus treatment (After Dara).

† The absolute cell count is performed by flow cytometry.

‡ Immunoglobulin is quantified by immunoturbidimetry.

Next, we conducted fiberoptic bronchoscopy, and subsequent bronchoalveolar lavage fluid (BALF) tested positive for MTB using nucleic acid amplification, together with CMV at a viral load of 2 × 10^5^ copies/mL. Microbial metagenomic next-generation sequencing (mNGS) of BALF also identified CMV and MTB with 4 reads and 344 reads, respectively. Moreover, the anti-acid stain of BALF showed positive results. Hence, we decided to postpone the lymphoma treatment till the TB infection was stabilized. He was then transferred to a tuberculosis dispensary, and a 4-drug regimen was initiated, including rifampicin 600 mg/d, isoniazid 300 mg/d, pyrazinamide 1500 mg/d, and ethambutol 750 mg/d. Ganciclovir 300 mg was administered simultaneously as an anti-CMV treatment twice a day. BALF yielded MTB colonies after 1-month culture, which confirmed the diagnosis of pulmonary TB.

After 2 months of oral anti-TB and antiviral therapy, the patient returned to our hospital for PBL treatment in June. On admission, he had normal vital signs, including temperature, and did not show any symptoms. PET/CT detected a partial response (Fig. 2). Laboratory examination revealed a normal blood count and ESR of 26 mm/h. Chest CT scan suggested improvement in the infection (Fig. 1). BALF and sputum smears were negative for MTB tests on three occasions, while CMV was still detectable. D-VCd treatment was administered concurrently with uninterrupted anti-TB and anti-CMV treatments with good tolerability. He completed the daratumumab-based therapy without the progression of the pulmonary infection. In September, he returned for PBL treatment while on continuous anti-TB treatment with no symptoms. Subsequently, the chest CT showed remarkable improvement in the infection (Fig. 1). Then, MTB was undetectable in BALF, and sputum smears samples, and the viral load of CMV declined to 10^3^ copies/mL in BALF. Abdominal CT suggested that lymphoma lesion was shrunk, and no monoclonal immunoglobulin was found in the blood serum or urine. The fifth course of daratumumab-based therapy was given as scheduled, and the patient was discharged with no symptoms.

## 3. DISCUSSION

Cellular and humoral immunity in patients with non-Hodgkin’s lymphoma frequently demonstrated hypogammaglobulinemia and, rarely, anergy.^12,13^ The treatments for lymphoma also affect the immune system.^14^ There are studies of immunologic function indicate defects in cell mediated immunity to herpes viruses for lymphoma patients treated with immunosuppressive agents.^15^ In general, a weakened immune system means vulnerable to infections and there is an increased risk of developing serious complications from infections.

To the best of our knowledge, this is the first example of the development of pulmonary TB infection and CMV reactivation during daratumumab-based therapy, indicating a possible association between antibody drug therapy and the infection. Typically, TB infections are described for patients with underlying disease and impaired immunity.^16^ Furthermore, studies have reported immune agents such as tumor necrosis factor-alpha (TNF-α) inhibitors, immune checkpoint inhibitors and ruxolitinib, could potentially increase the risk of TB infection.^17–19^ In this case, previous multiline treatments including bortezomib and cyclophosphamide, as well as daratumumab, may contribute to the possible immunosuppression and further cause TB infection.

Daratumumab targets CD38 expressed on the surface of many immune cells, including CD4+ T cell, CD8+ T cell, B lymphocytes, and natural killer cells.^20^ The application of CD38 antibody might influence these immune cells. Moreover, T cells are essential for TB prevention. Also, B cells and antibodies exert a protective role at each stage of TB infection.^21^ Thus, daratumumab treatment is a possible susceptibility factor in TB infection for this patient. The correlation between the administration of daratumumab and increased susceptibility to TB is yet to be explored.

The diagnosis of TB in patients with underlying diseases is challenging as they often present atypical clinical characteristics.^22^ For these patients, TB screening prior to treatment is crucial. CT examination and fiberoptic bronchoscopy are necessary to identify the pathogen.^23^ We conducted fiberoptic bronchoscopy and bronchoalveolar lavage for this patient prior to the next course of D-VCd, and found the specific pathogen, effectively avoiding serious complications.

Currently, there is no specific guideline to determine the duration and intensity of anti-TB treatment in this situation. Herein, we referred to clinicians’ experiences on immune checkpoint inhibitors^18^ and treated the patient with the standard combination of four medicines. After 6 months of treatment, the test results were negative, and the infection improved. The current case emphasizes that recognizing atypical symptoms of TB when administering the first-in-class human-specific anti-CD38 monoclonal antibody is essential. Still, additional studies are needed to define the characteristics and treatment of TB infection after daratumumab combination therapy.

CMV infection is a common problem for patients undergoing immunotherapy.^24^ The antiviral treatment is long-term. It is important to control the viral load and adjust the treatment timely.^25^ Ganciclovir is suitable for the patient in this case and the viral copy dropped dramatically after treatment. Meanwhile, the CMV titer of this patient was extremely high, which might affect the hemotopoiesis and lead to immunosuppression.

In conclusion, The current case demonstrated for the first time that TB infection and CMV reactivation might occur after daratumumab therapy. As CD38 monoantibody therapy becomes common, clinicians should be aware of the potential unusual infection and presentations. Routine TB and CMV screening might be considered prior to immunotherapy.

## ACKNOWLEDGMENTS

This work is supported by the fundings from the National Natural Science Foundation of China (81873427 and 82070217 to Dr. Jia Wei) and CHEN XIAO-PING Foundation for the Development of Science and Technology of Hubei Province (CXPJJH12000009-113, to Dr. Jia Wei).
